# Spontaneous contractions of the human thoracic duct—Important for securing lymphatic return during positive pressure ventilation?

**DOI:** 10.14814/phy2.15258

**Published:** 2022-05-17

**Authors:** Benjamin Kelly, Christopher L. Smith, Madhumitha Saravanan, Yoav Dori, Vibeke E. Hjortdal

**Affiliations:** ^1^ Department of Cardiothoracic and Vascular Surgery Aarhus University Hospital Aarhus Denmark; ^2^ Department of Clinical Medicine Aarhus University Aarhus Denmark; ^3^ Division of Cardiology Department of Pediatrics Children's Hospital of Philadelphia Philadelphia Pennsylvania USA; ^4^ Department of Cardiothoracic Surgery Rigshospitalet Copenhagen Denmark

**Keywords:** congenital heart defects, lymphatic contractions, lymphatic intervention, lymphatic physiology, positive pressure ventilation

## Abstract

The thoracic duct is responsible for the circulatory return of most lymphatic fluid. The return is a well‐timed synergy between the pressure in the thoracic duct, venous pressure at the thoracic duct outlet, and intrathoracic pressures during respiration. However, little is known about the forces determining thoracic duct pressure and how these respond to mechanical ventilation. We aimed to assess human thoracic duct pressure and identify elements affecting it during positive pressure ventilation and a brief ventilatory pause. The study examined pressures of 35 patients with severe congenital heart defects undergoing lymphatic interventions. Thoracic duct pressure and central venous pressure were measured in 25 patients during mechanical ventilation and in ten patients during both ventilation and a short pause in ventilation. TD contractions, mechanical ventilation, and arterial pulsations influenced the thoracic duct pressure. The mean pressure of the thoracic duct was 16 ± 5 mmHg. The frequency of the contractions was 5 ± 1 min^−1^ resulting in an average increase in pressure of 4 ± 4 mmHg. During mechanical ventilation, the thoracic duct pressure correlated closely to the central venous pressure. TD contractions were able to increase thoracic duct pressure by 25%. With thoracic duct pressure correlating closely to the central venous pressure, this intrinsic force may be an important factor in securing a successful return of lymphatic fluid. Future studies are needed to examine the return of lymphatic fluid and the function of the thoracic duct in the absence of both lymphatic complications and mechanical ventilation.

## Graphical abstract

In individuals with congenital heart defects undergoing lymphatic interventions, thoracic duct pressure correlates closely to central venous pressure. The thoracic duct spontaneously contracts with a frequency of 5 min^−1^ increasing thoracic duct pressure by 25%. Spontaneous contractions of the thoracic duct may be an important factor in securing the successful return of lymphatic fluid when challenged by positive pressure ventilation.

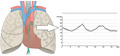

## 1 INTRODUCTION

Every day, an estimated 8 L of fluid is filtered from the circulation and out into the interstitial space (Levick & Michel, 2010). The lymphatic system takes up and removes this fluid, maintaining a fluid equilibrium. Through the lymphatic capillaries, the lymphatic collecting vessels, and ultimately the thoracic duct (TD), the fluid makes its way back into the venous circulation. Frequent lymphatic valves maintain unidirectional flow, while a pressure gradient drives forward the lymphatic fluid (Breslin et al., 2018). At the lympho‐venous junction, the fluid goes through the ostial valve which prevents blood from refluxing into the lymphatic system (El Zawahry et al., 1983).

The opening of the ostial valve is a well‐timed synergy between the pressure of the TD and the central venous pressure (CVP). The baseline of the CVP varies between individuals and cyclic fluctuations are determined mainly by cardiac pulsations and the intrathoracic changes in pressure during respiration. Similarly, the pressure in the TD is influenced by cardiac pulsations and changing pressures during respiration (Scallan et al., 2016; Schad et al., 1978), but in addition to these, the TD is also thought to rely on spontaneous contractions in order to increase pressure and drive forward lymphatic return. During mechanical ventilation, the thoracic duct return is faced with another challenge, as the habitual negative inspiratory airway pressure of −0.5 cmH_2_O is replaced with positive pressures of 20–30 cmH_2_O. Little is known about the role and influence of the contributing elements to thoracic duct pressure and how they are affected by the unphysiological changes during mechanical ventilation.

Individuals with congenital heart defects are often challenged by leakage of their lymphatic system. During lymphatic interventions, the thoracic duct is cannulated in order to locate and embolize these leaking lymphatic vessels. In addition, measurements of pressure are obtained to characterize the interplay between the pressure of the TD and the central veins. The goal of this study was to use these measurements to assess the forces influencing TD pressure during both mechanical ventilation and a short ventilatory pause.

## 2 METHODS

This is a retrospective review of measurements of TD pressure collected during lymphatic interventional procedures conducted at Children's Hospital of Philadelphia between February 2017 and April 2021. All measurements of TD pressure and CVP were collected routinely during interventions to evaluate thoracic duct patency and pressure. Included subjects were all surgically palliated with the Fontan operation for severe congenital heart defects leaving them with a univentricular circulation. All subjects were diagnosed with lymphatic complications: Plastic bronchitis, protein‐losing enteropathy, or chylothorax. To be included in this study, subjects needed to have recordings of the TD pressure and CVP measured at the same occasion available for comparison or to have had a short ventilatory pause during TD pressure measurement. Data collection included subject demographics, cardiac diagnoses, surgical history, lymphatic imaging, circulatory hemodynamics, ventilator settings, direct TD pressure measurements, and pressure waveform tracings. The institutional review board at Children's Hospital of Philadelphia approved the study and waived the need for informed consent (IRB 21/018765).

All procedures were performed under general anesthesia with neuromuscular blockade, endotracheal intubation, and positive pressure ventilation. For the lymphatic intervention, TD access was performed using inguinal intranodal lymphangiography and a transabdominal approach as previously described (Dori et al., 2016; Savla et al., 2017). Briefly, intranodal lymphangiography was performed using Lipiodol ethiodized oil (Gueber) to identify a target duct near the chylous cistern. Next, the TD was accessed transabdominally with a 22‐gauge Chiba needle (Cook Medical), V‐18 control wire (Boston Scientific), and Renegade microcatheter (Boston Scientific). Catheter position within the distal thoracic duct was confirmed using fluoroscopy and TD pressures were measured using the microcatheter or a RADI wire (Abbott Cardiovascular).

The waveform data were sampled with a custom program at 100 measurements/s. Data were exported to Microsoft Excel (Version 2016, Microsoft Corporation). All values were calculated as the average of five peaks/nadirs of TD contractions, ventilatory cycles, or heartbeats. The average pressure was calculated as the mean of five consecutive periods with an absence of TD contractions and ventilation. The average frequency was calculated by dividing the time (minutes) by the number of pressure peaks induced by contraction. A minimum of four contractions were used for each average frequency.

The data analyses for this study were conducted using Stata (Version 15.1, StataCorp). Data were checked for normality and reported as mean value ± SD or number (%). Pearson's correlation coefficient was used to test the correlation between TD pressure and CVP and between the relative increase in pressures and the positive inspiratory pressure. Simple linear regression was used to describe the association between changes in TD pressure and CVP and the positive inspiratory pressure.

Student's *t*‐test was used to test the hypothesis of no difference in CVP and TD pressure during inspiration and expiration and to test the hypothesis of no difference in the increase of CVP and TD pressure during positive‐pressure inspiration. Finally, Student's *t*‐test was used to test for difference between individuals with a positive and negative lymphovenous gradient with regard to CVP, TD pressure, and positive inspiratory pressure. A *p*‐value of <0.05 was considered significant.

## 3 RESULTS

Data were collected from 35 patients who had undergone a lymphatic intervention. Ten patients had measurements usable for evaluation of thoracic duct function during a brief ventilatory pause and 25 patients had pressure measurements of both TD pressure and venous pressure during mechanical ventilation. Measurements during the ventilatory pause were not standardized and varied in length from 40 to 50 s. Demographic information, circulatory information, lymphatic complications, findings on lymphatic and angiographic imaging, and planned lymphatic procedure are listed in Table 1.

**TABLE 1:** Demographic and clinical characteristics

	Ventilation (*n* = 25)	Pause (*n* = 10)
Demographic information		
Age, years	9 (±4)	11 (±6)
Female	11 (44%)	5 (50%)
BMI, kg/m^2^	18.2 (±5.2)	20.1 (±4.0)
Circulation		
Univentricular	25 (100%)	9 (90%)
Former univentricular	—	1 (10%)^a^
SVC and IVC patent	23 (92%)	10 (100%)
Lymphatic complication		
Plastic bronchitis	16 (64%)	4 (40%)
Protein‐losing enteropathy	10 (40%)	3 (30%)
Chylothorax	10 (40%)	6 (60%)
Lymphatic MR imaging (*n* = 23) (*n* = 10)^b^		
Pulmonary lymphatic perfusion pattern	19 (82.6%)	6 (60%)
Pulmonary effusion		
Right side	12 (53.2%)	6 (60%)
Left side	11 (47.8%)	5 (50%)
Pericardial effusion	3 (13%)	2 (20%)
Ascites	14 (60.9%)	6 (60%)
Lymphatic procedure		
Selective lymphatic duct embolization	19 (82.6%)	6 (60%)
Periduodenal and hepatoduodenal lymphatic embolization	3 (13%)	4 (40%)
Lymphatic catheterization	—	1 (10%)
Balloon dilatation angioplasty	—	2 (20%)
Innominate vein turn‐down	3 (13%)	—

Note

Data presented as *n* (%) or mean (±SD).

Abbreviations: BMI, Body mass index; IVC, Inferior vena cava; SVC, Superior vena cava.

^a^ One former univentricular patient had undergone biventricular repair.

^b^ Two patients in the ventilation group did not have a lymphatic MRI performed.

### 3.1 Components of thoracic duct pressure

**FIGURE 1:**
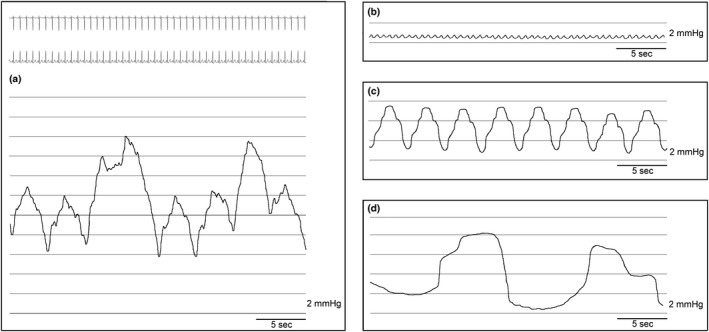
(a) Pressure measurement during mechanical ventilation including simultaneous cardiac tracing (top) (b) Isolated contribution from the cardiac pulsations with a heart rate of 92/min. (c) Isolated contribution from the mechanical ventilation with a ventilation rate of 18/min (d) Isolated contribution from the TD contractions with a contraction rate of 4/min. *(b), (c) and (d) are purely illustrative, and not actual recorded pressure tracings

From the pressure tracing (Figure 1a), three components of TD pressure could be identified. These were cardiac pulsations (Figure 1b), positive pressure ventilation (Figure 1c), and TD contractions (Figure 1d).

**FIGURE 2:**
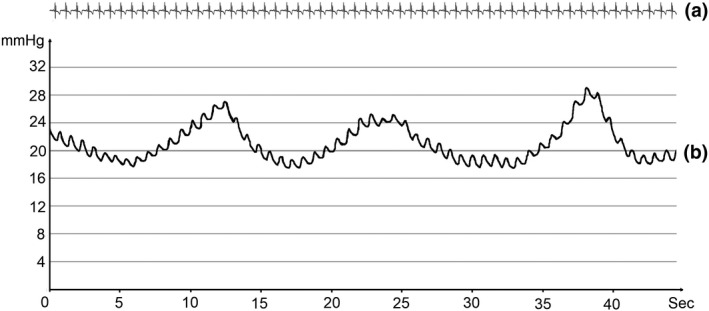
Pressure tracing during pause in ventilation. (a) Simultaneous cardiac tracing during pressure measurement. (b) Thoracic duct pressure tracing showing three contractions of the TD with a frequency of 4–5 min^−1^ and an up to 7–10 mmHg increase in pressure

All ten patients exhibited TD contractions during the ventilatory pause. The average contraction frequency was 5 min^−1^ (±1). There was some inter‐individual variation, with frequencies ranging between 3 and 7 min^−1^. The average increase in pressure generated by each contraction was 4 mmHg (±4) with increases spanning from 1 to 12 mmHg (See Figure 2).

**FIGURE 3:**
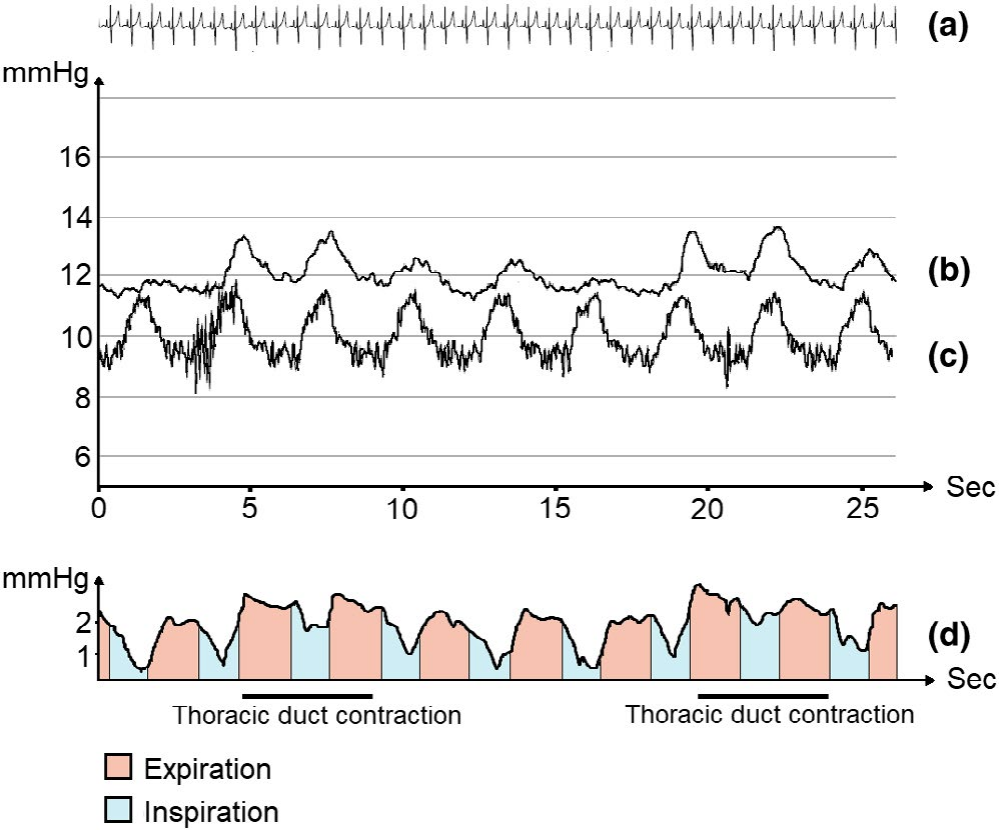
Pressure tracings during mechanical ventilation. *(*a) Simultaneous cardiac tracing during pressure measurement. (b) Thoracic duct pressure. (c) Central venous pressure. (d) Lymphovenous gradient during ventilation. Contractions of the thoracic duct predominantly affect the lymphovenous gradient during inspiration

During mechanical ventilation, we identified contractions in only 6 out of the same 10 subjects. From these limited observations, the contraction frequency during ventilation was 5 min^−1^ (±1) with an amplitude of 3 mmHg (±2) (See Figure 3). The average contribution to the TD pressure of each positive pressure inspiration was 3 mmHg (±2) ranging from 1 to 5 mmHg and the contribution from each cardiac pulsation was 0.7 mmHg (±0.5) ranging from 0.2 to 1.4 mmHg.

### 3.2 Impact of mechanical ventilation

In the ventilation group, measurements of CVP and the TD pressure were collected during both expiration and positive pressure inspiration. During the expiratory part of the breath cycle, the TD pressure was 16 ± 5 mmHg and the CVP was 16 ± 3 mmHg. Positive pressure ventilation significantly increased both TD pressure and venous pressure to 18 ± 5 mmHg and 19 ± 3 mmHg respectively (*p*‐value <0.001 for both comparisons). Positive pressure ventilation increased CVP by 3 ± 2 mmHg and TD pressure by 2 ± 2 mmHg, the venous pressure was significantly more affected than the TD pressure (*p*‐value = 0.001).

**FIGURE 4:**
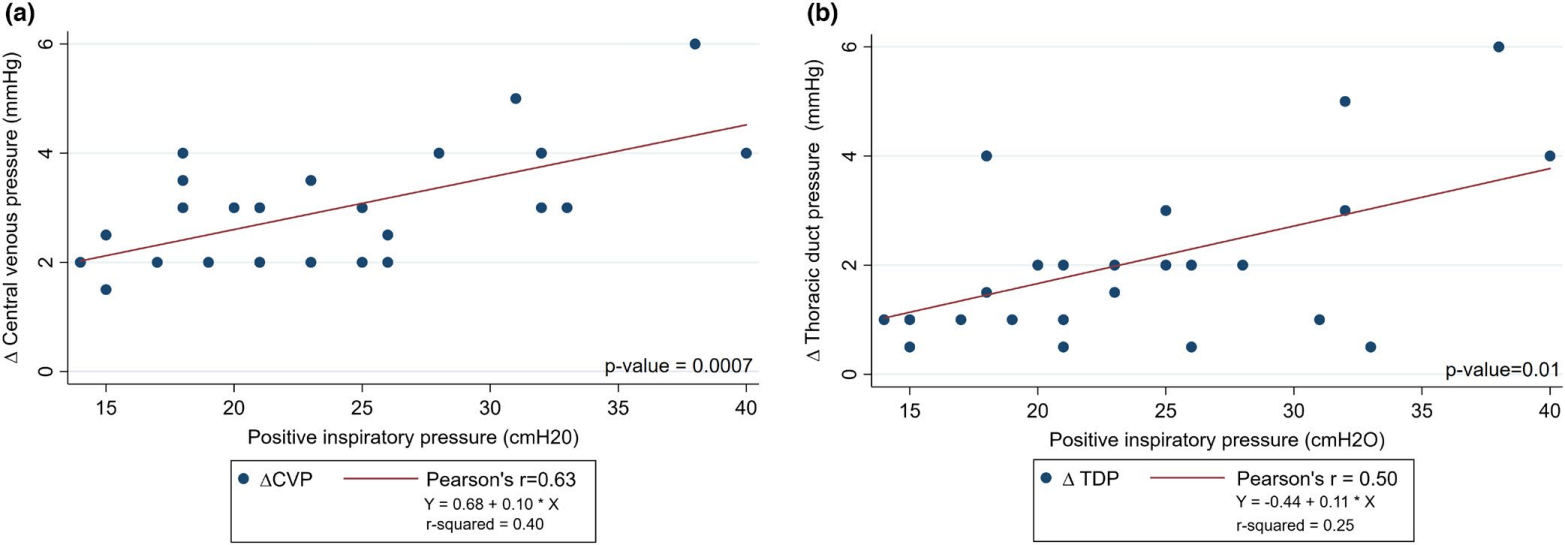
Scatter plot of the increase in pressure during inspiration and positive inspiratory pressure. The increase of both the CVP (a) and the TD pressure (b) correlated well with the size of the positive inspiratory pressure. CVP and TD pressure increased 0.10 mmHg and 0.11 mmHg respectively for each 1 cmH_2_O increase in positive inspiratory pressure. *(a) plot (21,2) and (b) plot (18,4) represent two observations each

The amplitudes of the increase in pressure of both the TD and the central venous pressure were closely correlated to the peak inspiratory pressure used for the ventilation. Increasing the peak inspiratory pressure increased the impact on CVP and TD pressure (See Figure 4). Pressures during ventilation and ventilator settings are listed in Table 2.

**TABLE 2:** Pressures and ventilator settings

	Ventilation (*n* = 25)
Pressure measurements	
Thoracic duct pressure, mmHg	
Expiration	16 (±5)
Inspiration	18 (±5)
Difference	2 (±2)
Central venous pressure, mmHg	
Expiration	16 (±3)
Inspiration	19 (±3)
Difference	3 (±1)
Ventilator settings	
Respiratory frequency, /min	16 (±4)
Peak inspiratory pressure, cmH_2_O	24 (±7)
Peak end‐expiratory pressure, cmH_2_O	2 (±2)
Mean airway pressure, cmH_2_O	7 (±2)

Note

Data presented as mean (±SD).

**FIGURE 5:**
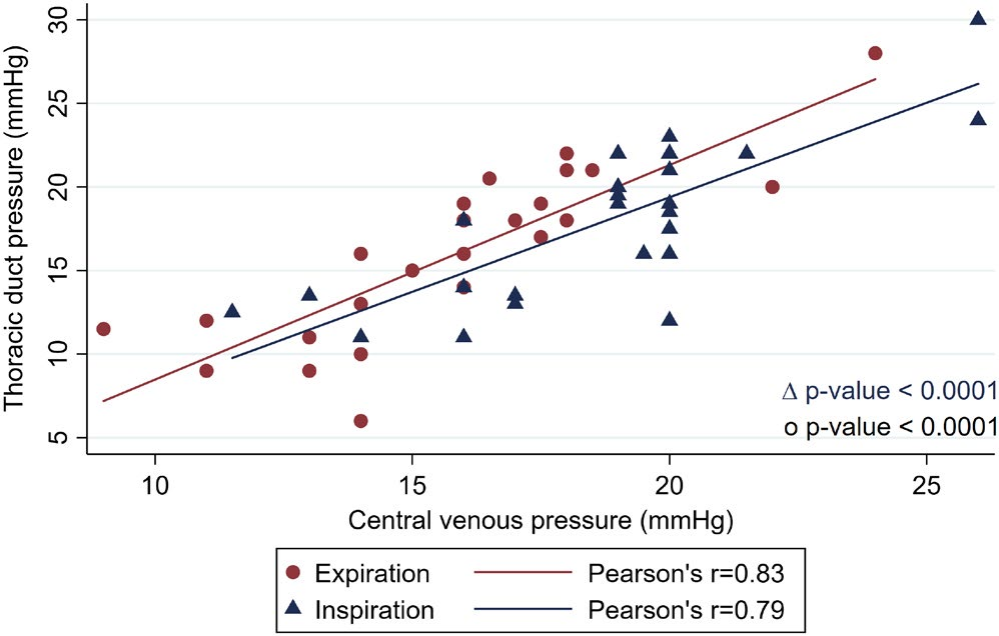
Scatter plot of TD pressure and CVP during inspiration (blue triangles) and expiration (red circles). The strongest correlation existed during expiration, where the impact of positive pressure ventilation was at a minimum. *plots at inspiration (20,22) and expiration (16,14) represent two observations each

TD pressure correlated significantly with the CVP during both expiration and positive pressure inspiration *r* = 0.83 and 0.79 respectively (*p*‐value <0.001 for both comparisons) (See Figure 5).

Of the examined individuals, 10 out of 25 had a negative lympho‐venous gradient during expiration and 12 out of 25 had a negative gradient during inspiration. Individuals with a negative gradient had a significantly lower TD pressure during both expiration 12 ± 4 versus 18 ± 4 mmHg (*p*‐value <0.01) and inspiration 15 ± 4 versus 20 ± 4 mmHg (*p*‐value <0.01) and were ventilated with a higher positive inspiratory pressure 27 ± 8 versus 21 ± 5 cmH_2_O (*p*‐value = 0.03) compared to individuals with a neutral or positive gradient. There was no difference when comparing CVP between the two groups during both inspiration and expiration (*p*‐value >0.36 for both comparisons).

## 4 DISCUSSION

In these minimally invasive pressure measurements, we report three elements to influence TD pressure: TD contractions, mechanical ventilation, and cardiac pulsations. Most prominent were the contractions increasing TD pressure by 25% at an average frequency of 5 min^−1^. The contractions were most notable during a short ventilatory pause. The TD pressure and CVP correlated closely during mechanical ventilation. The lympho‐venous gradient between the TD and the CVP varied in both size and direction between subjects.

Several earlier studies measuring TD pressure in humans have reported a spontaneous increase in pressure independent of ventilation and heart rate (Browse et al., 1974; Calnan et al., 1970; Edwards et al., 1965; Kinmonth & Taylor, 1956; Kinnaert, 1973; Pomerantz et al., 1963; Tilney & Murray, 1968). Similar to our findings during mechanical ventilation, the increases were not identifiable in all subjects (Kinnaert, 1973), which may explain why some studies also report no activity (Pomerantz et al., 1963). While the current study is limited to only measuring pressure, Kinmonth et al. found intra‐operative visual confirmation of TD contractions every 10–15 s (Kinmonth & Taylor, 1956). Considering these studies, we believe the observed increases in pressure to be caused by contractions of the TD. Perhaps due to the refined methods used and the short period of pause in ventilation, we identified contractions in all ten examined subjects, allowing us to ad detailed knowledge of the contraction frequency and the contractile function of the TD.

The ability of the TD to produce contractions is well known from ex‐vivo studies. Segments of the human TD can generate a 124% increase in pressure from 21 mmHg to 47 mmHg at an optimal passive stretch (Telinius et al., 2010). Additional stretching or relaxation of the vessel from this point decreases contractility. While contractions in the current study only increased pressure with an average of 25%, percentages ranged between 82.4% and 4.4%. Although preload, afterload, and type of lymphatic abnormalities may have varied between cases affecting contractility, we believe the 25% increase found in our study underlines a notable ability of the TD to contract and generate pressure.

The full mechanism behind the initiation and regulation of these contractions is incompletely understood. Apart from responding to mechanical stimuli such as stretch and flow over the lymphatic valves (Davis et al., 2012; Scallan et al., 2016), both local “pacemaker cells” (Briggs Boedtkjer et al., 2013) and adrenergic innervation (Telinius et al., 2013) are thought to be involved. In line with this complex regulation, the observed contractions were slightly irregular throughout measurements.

An influence from both mechanical ventilation and arterial pulsations on TD pressure has also been described sporadically in humans (Kinmonth & Taylor, 1956; Kinnaert, 1973; Tilney & Murray, 1968) and more detailed in canines (Browse et al., 1974; Pflug & Calnan, 1968). The main difference between mechanical ventilation and spontaneous respiration is the inspiratory decrease in venous pressure during spontaneous respiration and the rather large increase during positive pressure ventilation. The influence from arterial pulsations is minor and is believed to be transmitted to the TD from the aortic arch where the structures are in close proximity (Kinnaert, 1973).

The lymphatic return during mechanical ventilation probably differs from that during spontaneous respiration. The positive inspiratory pressure causes the lungs to expand and excerpt pressure on their surroundings. In the current population with venous congestion, the CVP and TD pressure were strongly proportional and correlated well. The CVP rose relatively more than TD pressure during positive‐pressure inspiration. Accordingly, when increasing positive inspiratory pressure with one cmH2o the rise in CVP was 10% greater than the rise in TD pressure challenging the lymphovenous gradient.

From a physiological point of view, a positive gradient between the TD and the central veins should open the ostial valve of the TD and cause movement towards a neutral gradient. Accordingly, the average gradient was 0 mmHg during expiration changing to −1 mmHg during inspiration. It is puzzling that the lympho‐venous gradient was negative throughout measurements in 10 out of 25 subjects. When examining this subgroup they had a significantly lower TD pressure with the average difference amounting to 4 mmHg while the CVP was similar between groups. In addition, subjects with an observed negative gradient were ventilated with a significantly larger positive inspiratory pressure, further challenging the maintenance of a favorable gradient for lymphatic return.

Considering the amounts of lymphatic fluid produced daily, a continuous negative gradient for lymphatic return should result in fluid accumulation and anasarca within days. However, the authors hypothesize on the following explanations: Due to their congenital heart defect and elevated CVP, the lymphatic vessels and TD of the examined subjects may be functionally altered (Mohanakumar et al., 2019, 2021). By working against an increased afterload their TD may have become first hypertrophic and then dilated impairing TD smooth muscle tone and function (Telinius et al., 2010). Consequently, new lympho‐venous connections may have become dominant in securing lymphatic return (Biko et al., 2019; Ghosh et al., 2020; Kelly et al., 2020). In addition, all subjects were undergoing lymphatic interventions meant to treat central lymphatic complications such as either plastic bronchitis or protein‐losing enteropathy. Here leakage from central lymphatic vessels is a central part of the pathophysiology (Dori et al., 2016; Rychik et al., 2020). In the case of a substantial leakage into low‐pressure environments such as airways or intestines, a low TD pressure also seems to be an option. Finally, it should be remembered that measurements were obtained during a limited period of mechanical ventilation. As discussed below, it is unknown if spontaneous respiration would have aided lymphatic return. In this case, excessive positive pressure ventilation could be thought to result in a universal accumulation of fluid, perhaps explaining the observation of the distal thoracic duct prolonged need for pleural drainage and increased net fluid balance following delayed extubation (Bianchi et al., 2021; Ovroutski et al., 2018).

During spontaneous respiration, little is known about the timing and cyclic nature of the lymphatic return. Exaggerated respiration increases filling and emptying of the TD and indicates respiration to be assisting lymphatic return (Pomerantz et al., 1963). Spanning both the thoracic and abdominal cavity, the extrinsic influence from respiration may differ depending on the cavity. While abdominal pressure rises during inspiration, thoracic pressure decreases potentially favoring a cephalad movement of lymphatic fluid. Contrarily, the decrease in abdominal pressure during expiration may allow the cisterna chyli to fill with lymphatic fluid from the periphery while the increase in pressure in the thoracic cavity may assist lymphatic fluid in overcoming venous pressure and returning to the circulation.

However, while the abdominal and thoracic pressures may assist fluid movement towards the ampulla of the TD, the exact timing of the return is filled with conflicting reports.

On one side, studies have reported valves opening (Riemenschneider & Shields, 1981; Seeliger et al., 1981) and greater flow of TD lymphatic fluid (Browse et al., 1974; Riemenschneider & Shields, 1981) during inspiration. Others, including a study using ultrasound on a similar cohort with congenital heart defects, found the lymphatic flow to be greatest during expiration (Kinnaert, 1973; Kochilas et al., 2014; Pflug & Calnan, 1968).

It remains unknown how the interplay between CVP and TD pressure would have been during spontaneous respiration. The mechanical ventilation and a study population with congenital heart defects and severe venous congestion may have affected findings. However, regardless of synchronization with the respiratory pressure changes, the 25% increase in pressure during contraction of the TD seems of such a magnitude, that it should be able to aid lymphatic return in most cases even when opposed by venous congestion and mechanical ventilation.

### 4.1 Limitations

The current method for pressure measurement was minimally invasive compared to previous methods (Kinnaert, 1973; Witte et al., 1969). The TD was cannulated in its abdominal part allowing a catheter to be advanced to the thoracic part for pressure sampling. The smooth muscle cells of the TD are a heterogeneous weaved net with circular, oblique, and longitudinal fibres (Telinius et al., 2010). Although less invasive, the abdominal cannulation may still have affected the contractile function of the TD further downstream in the thoracic cavity.

The lympho‐venous gradient should be interpreted with some caution. Some of the variations may be explained by fluctuation in pressure over time, as measurements in a few patients were not performed simultaneously. While a large positive lympho‐venous gradient may be explained by TD outlet occlusion, a continuous negative gradient remains a curiosity that warrants further investigation.

From a physiological point of view, measurements of the TD during spontaneous respiration of healthy subjects would be of the greatest interest. However, as the interventional lymphatic procedure requires patients to be anesthetized, this was not possible. Even so, we believe the results to aid in the understanding of the physiology of the lymphatic system.

## 5 CONCLUSION

Arterial pulsations, ventilation, and contractions affect TD pressure in patients with congenital heart defects undergoing lymphatic interventions. Most notably, the contractions were able to increase TD pressure by 25% at an average frequency of 5 min^−1^ in the absence of mechanical ventilation. With TD pressure correlating closely to the CVP, this intrinsic force may be an important factor in securing the successful return of lymphatic fluid. Future studies need to illuminate the return of lymphatic fluid and TD function during spontaneous respiration.

## CONFLICT OF INTEREST

None.

## ETHICS STATEMENT

The institutional review board at Children's Hospital of Philadelphia approved the study and waived the need for informed consent (IRB 21/018765).

## AUTHOR CONTRIBUTION

BK, CS, MS, YD VEH contributed to the conception and design of the study. MS and BK collected data and analyzed data. BK, CS, MS, YD VEH all contributed to interpretation, drafting and revision of the article. All authors approved the final version prior to publication.
